# Assessing Drought and Heat Stress-Induced Changes in the Cotton Leaf Metabolome and Their Relationship With Hyperspectral Reflectance

**DOI:** 10.3389/fpls.2021.751868

**Published:** 2021-10-22

**Authors:** Giovanni Melandri, Kelly R. Thorp, Corey Broeckling, Alison L. Thompson, Lori Hinze, Duke Pauli

**Affiliations:** ^1^School of Plant Sciences, University of Arizona, Tucson, AZ, United States; ^2^United States Department of Agriculture-Agricultural Research Service, Arid Land Agricultural Research Center, Maricopa, AZ, United States; ^3^Analytical Resources Core: Bioanalysis and Omics Center, Colorado State University, Fort Collins, CO, United States; ^4^Department of Agricultural Biology, Colorado State University, Fort Collins, CO, United States; ^5^United States Department of Agriculture-Agricultural Research Service, Southern Plains Agricultural Research Center, College Station, TX, United States

**Keywords:** cotton, drought, heat, leaf metabolome, hyperspectral reflectance, partial least squares regression

## Abstract

The study of phenotypes that reveal mechanisms of adaptation to drought and heat stress is crucial for the development of climate resilient crops in the face of climate uncertainty. The leaf metabolome effectively summarizes stress-driven perturbations of the plant physiological status and represents an intermediate phenotype that bridges the plant genome and phenome. The objective of this study was to analyze the effect of water deficit and heat stress on the leaf metabolome of 22 genetically diverse accessions of upland cotton grown in the Arizona low desert over two consecutive years. Results revealed that membrane lipid remodeling was the main leaf mechanism of adaptation to drought. The magnitude of metabolic adaptations to drought, which had an impact on fiber traits, was found to be quantitatively and qualitatively associated with different stress severity levels during the two years of the field trial. Leaf-level hyperspectral reflectance data were also used to predict the leaf metabolite profiles of the cotton accessions. Multivariate statistical models using hyperspectral data accurately estimated (*R*^2^ > 0.7 in ∼34% of the metabolites) and predicted (*Q*^2^ > 0.5 in 15–25% of the metabolites) many leaf metabolites. Predicted values of metabolites could efficiently discriminate stressed and non-stressed samples and reveal which regions of the reflectance spectrum were the most informative for predictions. Combined together, these findings suggest that hyperspectral sensors can be used for the rapid, non-destructive estimation of leaf metabolites, which can summarize the plant physiological status.

## Introduction

Drought and heat are two major abiotic stresses that often occur in the field simultaneously and whose effects negatively impact crop growth and productivity (Mittler, 2006; Suzuki et al., 2014). In the last decades, the frequency of drought and heat stress events has increased as a result of global climate change posing a threat to present and future crop production (Zscheischler et al., 2018; Alizadeh et al., 2020). In the face of these challenges, the ability to identify key mechanisms of adaptation to heat and drought stress is pivotal for improving plant resiliency and maintaining crop productivity.

Upland cotton (*Gossypium hirsutum* L.) is a crop of primary importance in Arizona, with ∼50,000 ha planted in 2020 and an annual production value of $110 million for both fiber and seeds (USDA, 2021). The primary cotton production system in the Arizona low desert uses surface irrigation to supplement limited precipitation. When cotton is grown in semi-arid environments, well above the thermal optimum of 30/22°C day/night temperature (Burke and Wanjura, 2010), water availability is also associated with heat stress avoidance through transpiration-driven leaf cooling that, on very hot days, can reach 10°C relative to ambient atmospheric temperatures (Burke and Upchurch, 1989; Carmo-Silva et al., 2012). In this context, the ongoing drought in the Colorado River basin, the main source of irrigation water for Arizona growers, and the projected increase in temperature due to global climate change are estimated to cause ∼40 and ∼50% reduction in cotton yield by mid- and late-century, respectively (Ayankojo et al., 2020; Thorp et al., 2020). For these reasons, is necessary the development of new cotton accessions more adapted to heat and drought stress.

The plant metabolome represents the summation of numerous metabolic pathways modulated by intracellular and intercellular regulatory processes, and it incorporates the effect of genetic (i.e., pleiotropy and epistasis) and environmental factors, as well as their interactions (Keurentjes, 2009; Herrmann and Schauer, 2013). Mechanisms of plant adaptation to heat and drought conditions involve morphological, physiological, and molecular responses that ultimately lead to metabolic changes (Fahad et al., 2017; Zandalinas et al., 2018). Under stressful conditions, the reprogramming of plant metabolism is characterized by a trade-off between growth and survival during which the activity of essential metabolic pathways needs to be maintained while adapting to the new prevailing environmental conditions (Obata and Fernie, 2012; Claeys and Inzé, 2013). Drought and heat stress-induced metabolic changes have been extensively studied in many plant species and include, among others, an overall accumulation of amino acids and sugars associated with reduced growth, an increase in compatible solutes with protective functions (e.g., betaines, trehalose, raffinose, and proline), and membrane lipid remodeling (Krasensky and Jonak, 2012; Obata and Fernie, 2012; Liu et al., 2019). A number of studies have specifically investigated the combined effect of heat and drought stress on the metabolite profile of plants (Rizhsky et al., 2004; Obata et al., 2015; Templer et al., 2017). These studies highlighted that the combination of the two stresses induced only a few additional specific responses while most of the metabolic changes were caused by the sum of the individual stress responses, with drought having the strongest effect. Because of their strong relationship with plant physiological status, metabolites are regarded as functional intermediate phenotypes more closely linked to complex plant traits (e.g., drought tolerance) than, for example, gene transcripts (Fiehn, 2002; Luo, 2015). For these reasons, multivariate analysis of plant metabolism has shown a great potential for the prediction of complex phenotypic traits and selected metabolites have been considered as promising targets for crop improvement (Razzaq et al., 2019; Melandri et al., 2020; Fernandez et al., 2021). Despite their potential, a main limitation to the use of metabolites in breeding depends on the time and costs required for their extraction and quantification. A possible solution to this problem is offered by the technological developments in field-based, high-throughput plant phenotyping, which has enabled rapid and low-cost measurements of many plant phenotypes across time and space (Pauli et al., 2016b; Araus et al., 2018). Particularly, several studies have demonstrated that the collection of hyperspectral proximal sensing data (with spectral range of 350–2500 nm) of plant canopies has enabled rapid and non-destructive estimation (by multivariate statistical modeling) of leaf chemical properties including photosynthetic pigments as well as nitrogen, potassium, phosphorus, and lignin content in many plant species (Martin et al., 2008; Kokaly et al., 2009; Asner et al., 2011; Pimstein et al., 2011). More recently, Vergara-Diaz et al. (2020) were able to predict metabolite values in flag leaves and ear bracts of durum wheat using hyperspectral reflectance data collected at leaf and canopy level. In the near future, routine use of high-throughput hyperspectral sensors for the remote and non-destructive estimation of plant metabolites is possible, which could enable biochemical phenotyping of plants in a time- and cost-efficient manner (Burnett et al., 2021). However, additional studies are necessary to evaluate the accuracy of the relationship between hyperspectral data and leaf metabolites in a larger number of the crop species and to test their robustness under different environmental conditions.

The goal of this study was to evaluate the relationship between leaf metabolite profiles and leaf-level spectral reflectance in 22 genetically diverse accessions of upland cotton grown under well-watered (WW) and water-limited (WL) conditions in the Arizona low desert over two consecutive years. Specific objectives were to (1) quantify impacts of heat and drought stress on the cotton leaf metabolome and (2) evaluate leaf reflectance spectra for estimating metabolite profiles of stressed and non-stressed cotton leaves.

## Materials and Methods

### Plant Material, Experimental Design, and Soil Measurements

A panel of 22 cotton (*Gossypium hirsutum* L.) accessions (Supplementary Table 1) were evaluated in the years 2018 and 2019 at the Maricopa Agricultural Center (MAC) in Maricopa, AZ, United States (33°04′37″N, 111°58′26″W, elevation 358 m). The experimental field site of this study, located in the low desert of central Arizona, provided optimal meteorological conditions for studying the response of plants to the effect of heat and drought stress. The panel consisted of three commercial check accessions (FiberMax 958, DeltaPine 493, and DeltaPine 393) and 19 accessions from the Gossypium Diversity Reference Set (Percy et al., 2014). The accessions of the panel were chosen to provide a representation of the genetic diversity contained within the entire GDRS collection. The 22 accessions were evaluated under two irrigation treatments, WL and WW, at a field site with soil characterized as Casa Grande sandy loam (fine-loamy, mixed, superactive, and hyperthermic Typic Natrargids). Each year the accessions were arranged in a randomized incomplete block design with two replications per irrigation treatment totaling 88 experimental plots. Experimental plots were 4.50 m in length with a 1.21-m alley at the end and were seeded at a rate of ∼12 plants per linear meter with an inter-row spacing of 1.02 m. Conventional cotton cultivation practices for the desert Southwest were employed. Meteorological data were obtained from an automated Arizona Meteorological Network (AZMET) weather station^1^ located on the research facility (Brown, 1989).

The crop was established using a variable-rate, overhead linear move irrigation system (Lindsay Corporation, Omaha, Nebraska) with 0.201 L s^–1^ nozzles (#13.5, Senninger, Clermont, FL, United States). Once plants had emerged and developed four true leaves, neutron moisture probe access tubes were placed to a depth of 2.00 m in the middle of each irrigation treatment area (comprised of 44 plots) using a tractor-mounted soil sampler (Model 25-TS, Giddings Machine Company, Windsor, CO, United States). Measurements of soil water content were collected on a weekly basis at six depths (10, 30, 50, 70, 90, and 110 cm) using a field-calibrated neutron moisture probe (Model 503, Campbell Pacific Nuclear, CPN, Martinez, CA, United States). The scheduling of the WW irrigation treatment was specified based on simulations from the CSM-CROPGRO-Cotton model, following the methodology of Thorp et al. (2017). The model computed a daily soil water balance crop evapotranspiration (ETc) based on the FAO-56 dual crop coefficient method (DeJonge and Thorp, 2017), and cotton growth, stress, and yield metrics. Irrigation amounts were determined as the smallest rate that supplied model-predicted cumulative seasonal ETc. This eliminated predictions of water stress and maintained root-zone soil water depletion below 45%. Further irrigation management details were provided by Thorp et al. (2020). The WL irrigation treatment was initiated when 50% of the plots were at first flower (on Julian days 201 and 192 in 2018 and 2019, respectively), after which this treatment received half of the irrigation amount computed for the WW treatment. Irrigation rates for the WL treatment were applied using the variable-rate irrigation system to reduce application rates over the WL treatment area. Scheduling of irrigation was performed on a weekly basis by incorporating updated daily meteorological information, including best estimates of future conditions.

At the end of each growing season, 25 bolls were randomly sampled (on Julian days 290 and 288 in 2018 and 2019, respectively) from each plot and processed using a laboratory 10-saw gin to collect fiber for the analysis of their quantity and quality. Lint yield (grams/plot) after gin was measured at MAC. Fiber quality measurements were made using an Uster HVI 1000 (High Volume Instrument, Uster, Charlotte, NC, United States) at Cotton Incorporated (Cary, NC, United States). The fiber quality traits measured were micronaire (Mic, units of air permeability), upper-half mean length (UHM, inches), length uniformity (UI, percent), strength (Str, grams per tex), and elongation (Elo, percent).

### Tissue Sampling

Leaf sample collection occurred on Julian day 239 (at flowering/boll development stage) in both years and began at 10:30 in the WW treatment with collection of samples from the WL treatment beginning at approximately 12:30 and concluding at approximately 14:30. Leaf tissue samples (two disks per leaf) were collected from the upper-most expanded leaf of five randomly selected plants within each plot. The two, 0.64-cm diameter, leaf disks were collected with a leaf punch (J Tissue Punch, Midco Global, Kirkwood, MO, United States). The punched leaf tissue was directly sampled into a 2 mL microfuge tube that was prefilled with 1 mL of reagent-grade methanol (Sigma Aldrich, St. Louis, MO, United States) and kept on ice. While in the field, collected samples were stored on ice in coolers. When sampling was complete, samples were moved inside to a −80°C freezer for storage. Samples were then transferred to a 2 mL polypropylene screw top tube (Corning Inc., Corning, NY, United States) and shipped overnight on dry ice to the Analytical Resources Core: Bioanalysis and Omics Center at Colorado State University (Fort Collins, CO, United States).

### Hyperspectral Data Collection

Radiometric measurements of cotton leaves were collected within each experimental plot using a portable field spectroradiometer (ASD FieldSpec 3, Malvern Panalytical, Inc., Westborough, MA, United States). Radiometric information was reported in 2151 narrow wavebands from 350 to 2500 nm with bandwidth of 1 nm. The instrument’s 1-m fiber optic cable was fitted with a contact probe and a leaf clipping device for non-destructive, *in situ* radiometric measurements of cotton leaves. The probe featured a 4.5-W halogen light source and measured leaf radiance independent of external lighting conditions. Two reflectance standards (one made of white polytetrafluoroethylene material and the other made of black painted vinyl) were incorporated with the leaf clip and were easily interchanged to alternate between white reference measurements and dark background for leaf measurements. Spectral measurements of the five leaves per plot were collected immediately prior to sampling tissue from those leaves. Ten spectral measurements were collected from each leaf and averaged by the instrument’s control software; the averaged spectra were saved to one file per leaf. Five spectral scans of the white reference panel were collected to characterize the light provided by the halogen bulb bookending the spectral measurements for groups of eight plots. Following spectral data collection, leaf reflectance factors were computed as the ratio of leaf radiance and the radiance data from the previous white reference scan. Reflectance data from the five leaves per plot were averaged to provide plot-level spectra.

### Metabolite Extraction and Quantification

Leaf tissue samples were extracted in 100% methanol using a biphasic protocol (Matyash et al., 2008). For each sample, the content of the 2 mL polypropylene tube (leaf discs and methanol) was transferred to a 22 mL glass vial. The original tubes were rinsed with methanol that was also transferred to the same extraction vial, resulting in a 4 mL volume of methanol per sample. The glass vial was sonicated for 30 min in an ice bath, followed by 2 h of vigorous mixing at 4°C. Samples were dried by removing the solvent under nitrogen gas. The dried samples were re-suspended by adding 3 mL of ice-cold methanol and mixing for 10 min at 4°C. Next, 1 mL of water, and 6 mL of methyl tert-butyl ether (MTBE) were added to the re-suspended samples that were sonicated for 30 min and mixed for 2 h at 4°C. To induce phase separation, 4 mL of water was added to each sample. After 30 min of mixing at 4°C followed by centrifugation at 2500 × *g* for 15 min at 4°C, 400 μL of the upper organic phase was transferred to a new vial, dried under nitrogen, and re-suspended in 100 μL of methanol/toluene (1:1, by volume) for liquid chromatography–mass spectrometry (LC–MS) analysis. A 200 μL aliquot of the lower aqueous phase was dried under nitrogen and stored at −80°C until gas chromatography–mass spectrometry (GC–MS) analysis. Quality control (QC) samples for LC–MS and GC–MS analyses were generated by pooling the organic and aqueous extracts of each sample, respectively.

To conduct the GC–MS metabolite analysis, dried samples were re-suspended in 50 μL of pyridine containing 25 mg mL^–1^ of methoxyamine hydrochloride, incubated at 60°C for 1 h, sonicated for 10 min, and incubated for an additional 1 h at 60°C. Next, 50 μL of *N*-methyl-*N*-trimethylsilyltrifluoroacetamide with 1% trimethylchlorosilane (MSTFA + 1% TMCS, Thermo Scientific) was added and samples were incubated at 60°C for 45 min, briefly centrifuged, cooled to room temperature, and 80 μL of the supernatant was transferred to a 150 μL glass insert in a GC–MS autosampler vial. Metabolites were detected using a Trace 1310 GC coupled to a Thermo ISQ mass spectrometer (Thermo Scientific). One microliter of each sample was injected in a 1:10 split ratio in randomized order. Separation was achieved using a 30 m TG-5MS column (Thermo Scientific, 0.25 mm i.d., 0.25 μm film thickness) with a 1.2 mL min^–1^ helium gas flow rate with the following steps: 80°C for 30 s, a ramp of 15°C per min to 330°C, and an 8 min hold. Masses between 50 and 650 m/z were scanned at 5 scans per s after electron impact ionization. QC samples were injected after every six samples. For each sample, the raw data files from the mass spectrometer were converted to.cdf format, and a matrix of molecular features as defined by retention time and mass (m/z) was generated using XCMS software in R (Smith et al., 2006) for feature detection and alignment using the matchedFilter algorithm. Features were grouped using RAMClustR (Broeckling et al., 2014), with normalization set to “TIC.” GC–MS spectra were annotated by matching unknown spectra to the Golm metabolome retention indexed spectral library (Kopka et al., 2005), using retention times plotted vs. the Golm retention index to increase confidence in the spectral match. Searching was accomplished using the RAMSearch program (Broeckling et al., 2016). Additional GC–MS matching was performed by searching against the NIST v12 EI spectral database. All data analyses were performed using R (R Core Team, 2020).

To conduct the LC–MS metabolite analysis, 1 μL of the re-suspended extract was injected onto a Waters Acquity UPLC system in randomized order and separated in a Waters Acquity UPLC CSH Phenyl Hexyl column (1.7 μM, 1.0 mm × 100 mm), using a gradient from solvent A (Water, 0.1% formic acid) to solvent B (Acetonitrile, 0.1% formic acid). Injections were made in 99% A, held at 99% A for 1 min, ramped to 98% B over 12 min, held at 98% B for 3 min, and then returned to starting conditions over 0.05 min and allowed to re-equilibrate for 3.95 min, with a 200 μL min^–1^ constant flow rate. The column and samples were held at 65 and 6°C, respectively. The column eluent was infused into a Waters Xevo G2 Q-TOF-MS with an electrospray source in positive mode, scanning 50–2000 m/z at 0.2 s per scan, alternating between MS (6 V collision energy) and MSE mode (15–30 V ramp). Calibration was performed using sodium iodide with 1 ppm mass accuracy. The capillary voltage was held at 2200 V, source temperature at 150°C, and nitrogen desolvation temperature at 350°C with a flow rate of 800 L h^–1^. QC samples were injected after every 6 samples. LC–MS data were first annotated by searching against an in-house spectra and retention time database using the RAMSearch program (Broeckling et al., 2016). RAMClustR (Broeckling et al., 2014) was used to call the function *findMain* (Jaeger et al., 2017) from the R package “interpretMSSpectrum” to infer the molecular weight of each LC–MS compound and annotate the mass signals. The complete MS spectrum and a truncated MSE spectrum were converted to a.mat format for import into MSFinder (Tsugawa et al., 2016). The MSE spectrum was truncated to only include masses with values less than the inferred M plus its isotopes, and the.mat file precursor ion was set to the M + H ion for the findMain inferred M value. These.mat (file format) spectra were analyzed to determine the most probable molecular formula and structure. MSFinder was also used to perform a spectral search against the MassBank database. All results were imported into R and a collective annotation was derived with prioritization of RAMSearch > MSFinder mssearch > MSFinder structure > MSFinder formula > findMain M. Annotation confidence was set as described by Sumner et al. (2007). All data analyses were performed using R (R Core Team, 2020). The signal intensity of a compound showing multiple ions was calculated considering the weighted mean (based on mean intensity in all samples) of all the single ions. Only compounds annotated with an International Chemical Identifier Key (InChIKey) were considered for all the further statistical analyses.

### Pre-processing of Fiber, Metabolic, and Spectral Data

The values of each metabolite quantified in 1 year using GC–MS and LC–MS were rescaled (division) using the estimated median of the metabolite in that specific year. This preprocessing step was necessary to reduce the computational cost of the error variance prediction using the mixed linear model described below.

For fiber yield/quality data, metabolites, reflectance spectra, and vegetation indices (VIs), the Box–Cox power transformation (Box and Cox, 1964) was performed on raw phenotypic data (fiber yield/quality, spectra, and VIs) or on median-rescaled values (metabolites) using a simplistic linear model that included genotype and treatment as fixed effects to identify the most appropriate transformation, if required. These transformations were to correct for non-normality of the error terms and unequal variances with respect to the individual traits. The procedure evaluated lambda values ranging from −2 to +2 in increments of 0.5 using the function *boxcox* in the R package “MASS” (Venables and Ripley, 2002) followed by applying the optimal convenient lambda for each individual trait.

To identify and remove statistically significant outliers, a mixed linear model was fitted for each phenotypic trait within each year (i.e., years were analyzed individually) using ASReml-R version 3.0 (Gilmour et al., 2009). The full model (Equation 1) fitted to the phenotypic data that had been processed by the Box–Cox transformation procedure was:

(1) $$\begin{array}{c} {\text{Y}}_{ijklm}=\text{μ}+{\text{genotype}}_{i}+{\text{irg}}_{j}+\text{genotype}\times {\text{irg}}_{ij}\\ +\text{rep}(\text{irg}{)}_{kj}+\text{block}(\text{rep}\times \text{irg}{)}_{jkl}\\ +\text{column}(\text{rep}\times \text{irg}{)}_{jkm}+{\text{ε}}_{ijklm} \end{array}$$

where Y*_*ijklm*_* is an individual phenotypic observation; μ is the experimental grand mean; genotype*_*i*_* is the effect of the *i*-th genotype (accession); irg*_*j*_* is the effect of the *j*-th irrigation treatment which was either WW or WL; genotype × irg*_*ij*_* is the interaction effect between the *i*-th genotype and the *j*-th irrigation treatment; rep(irg)*_*kj*_* is the effect of the *k*-th replication nested within the *j*-th irrigation treatment; block(rep × irg)*_*jkl*_* is the effect of the *l*-th block nested with *k*-th replication within the *j*-th irrigation treatment; column(rep × irg)*_*jkm*_* is the effect of the *m*-th plot grid column nested within the *k*-th replication within the *j*-th irrigation treatment; and ε*_*ijklm*_* is the residual error term assumed to be independently and identically distributed according to a normal distribution with mean zero and variance $\sigma_{\varepsilon }^{2}$. The model terms μ, genotype*_*i*_*, irg*_*j*_*, and genotype × irg*_*ij*_* were modeled as fixed effects with all remaining terms being modeled as random effects. Degrees of freedom were calculated *via* the Kenward–Rogers approximation (Kenward and Roger, 1997). The Studentized deleted residuals (Neter et al., 1996) obtained from the fitted mixed linear models were examined to detect and remove significant outliers.

Once outliers for all phenotypic traits were removed, an iterative mixed linear model fitting procedure was conducted in ASReml-R version 3.0 using Equation 1 as the full model (Gilmour et al., 2009). To derive the best fitted model for each individual trait, likelihood ratio tests were carried out to remove all random effects that were not significant at the α = 0.05 (Littell et al., 2006). The final fitted model for each individual trait was used to generate a best linear unbiased estimator (BLUE) for each genotype.

For each trait, repeatability (*r*) was calculated to express the proportion of variance due to permanent, non-localized differences (i.e., not due to experimental error) between genotypes to provide a measure of technical performance. Using Equation 1, the model was reformulated so that all terms were modeled as random effects to derive the respective variance components (Equation 2). The variance component estimates from the full model were used to estimate *r* as follows:

(2) $$r=\frac{{\hat{\sigma }}_{g}^{2}}{{\hat{\sigma }}_{g}^{2}+\frac{{\hat{\sigma }}_{g\,i}^{2}}{n_{i\,r\,g}}+\frac{{\hat{\sigma }}_{\varepsilon }^{2}}{n_{p\,l\,o\,t}}}$$

where ${\hat{\sigma }}_{g}^{2}$ is the estimated variance due to the genotypes, ${\hat{\sigma }}_{g\,i}^{2}$ is the estimated variance associated with the genotype-by-irrigation treatment variation, and ${\hat{\sigma }}_{\varepsilon }^{2}$ is the residual error variance. The variable *n*_*irg*_ is the harmonic mean of the number of irrigation treatments in which each genotype was observed and *n*_*plot*_ is the harmonic mean of the number of plots in which genotype was observed. It should be noted that the denominator of Equation 2 is equivalent to the phenotypic variance, $\hat{\sigma_{p}^{2}}$, of a trait. Standard errors of the estimated repeatability for each individual trait were approximated using the delta method (Lynch and Walsh, 1998; Holland et al., 2010).

For those individual traits which the Box–Cox procedure indicated a transformation was needed, the inverse of the convenient lambda used was applied to back-transform the estimated BLUEs. The back-transformed BLUEs of metabolites were then rescaled by multiplying their values by the median value determined in the initial processing steps, and the values were used for all subsequent statistical analyses. Repeatability values and fixed effects’ *P*-values determined by the mixed linear models for the fiber yield/quality in 2018 and 2019 are reported in Supplementary Tables 2, 3. The same summary for metabolites showing a significant (*P* < 0.05) change due to the effect of the irrigation treatment in 2018 and 2019 (considered as the starting dataset in all the following statistical analyses) are reported in Supplementary Tables 4, 5 and the summary for the VIs in 2018 and 2019 is reported in Supplementary Table 6. The BLUEs of single accessions in the 2 years of the field experiment for all the fiber yield/quality data, metabolites, hyperspectral data, and VIs are provided in Supplementary Data 1.

### Statistical Analysis of Metabolite Data

All statistical analyses were performed using R statistical software (R Core Team, 2020). Imputation of missing metabolite values, prior to any other statistical test, was performed by the function *knnImputation* in the R package “DMwR” (Torgo, 2010). Principal component analysis (PCA) was conducted to assess the overall effect of treatment on the leaf metabolic profile of the accessions. PCA was performed on log_10_ transformed (to improve normality), centered (mean subtraction) and scaled (standard deviation division) metabolite data using the function *prcomp* in the R package “stats.” Hierarchical clustering analysis was conducted to evaluate the effect of stress-induced leaf metabolic changes across the two years. For this purpose, only metabolites identified in common between the two years were used in the analysis. To overcome the analytical differences in the quantification of these metabolites over the two years, their values were median-normalized (Lisec et al., 2011; Lawas et al., 2019) by multiplying each metabolic value by a correction factor. This factor was calculated as the median value of the metabolite across the samples of the two years divided by the median value of the same metabolite among the samples of the specific year (2018 or 2019). Median-normalized metabolic values were then log_10_ transformed, centered, and scaled before being analyzed by hierarchical clustering analysis that was performed and visualized (dendrograms and heatmap) using the function *pheatmap* in the R package “pheatmap” (Kolde, 2012) using Pearson correlation as clustering distance and complete linkage as clustering method.

Stress-induced mean fold change (FC) of each metabolite was calculated (on non-log-transformed data) dividing the mean value of the 22 accessions under WL conditions by the same value under WW conditions. A mean FC threshold (FC <0.5 or >2) was applied to select metabolites with high mean deviation from non-stressed conditions. Significant differences in metabolite levels between the group of cotton accessions grown under WL and WW conditions were further tested by the non-parametric two-sided Mann–Whitney *U*-test using the function *wilcox.test* in the R package “stats.” Mann–Whitney *U*-test’s *P*-values were corrected for multiple testing according to Benjamini and Hochberg (1995) with a false discovery rate (FDR) of 0.05 using the function *p.adjust* in the R package “stats.” Volcano plots of metabolites based on stress-induced mean FC (log_2_-scaled) and Mann–Whitney *U*-test’s *P*-values (−log_10_-scaled) were generated using the function *EnhancedVolcano* in the R package “EnhancedVolcano” (Blighe et al., 2018).

### Vegetation Indices

Vegetation indices that estimate leaf physiological and water status conditions were calculated based on specific reflectance wavelengths (*R*_*x*_). The normalized difference vegetation index (NDVI) was calculated as in Pauli et al. (2016a): (*R*_820_ − *R*_670_)/(*R*_820_ + *R*_670_). The photochemical reflectance index (PRI) was calculated as in Gamon et al. (1997): (*R*_531_ − *R*_570_)/(*R*_531_ + *R*_570_). Scaled photochemical reflectance index (sPRI) was then calculated, to avoid negative values of PRI, as in Letts et al. (2008): (PRI + 1)/2. The carotenoids reflectance index (CRI) was calculated as in Gitelson et al. (2002): (*R*_510_)^–1^/(*R*_550_)^–1^. The ratio between the water index (WI) and NDVI (WI/NDVI) was calculated as in Penuelas et al. (1997) and in Penuelas and Inoue (1999): (*R*_900_ − *R*_970_)/NDVI. The ratio normalizes WI for structural and color changes detected by NDVI in the drying leaves thus maximizing the sensitivity of the index to water content.

### Hyperspectral-Based Partial Least Squares Models for the Estimation and Prediction of Metabolites

Partial least squares regression (PLSR) modeling was used to relate the leaf spectral measurements with the metabolite data. Thorp et al. (2011) provided the details on the PLSR methodology used herein. Briefly, if **Y** is an *n* × 1 vector of responses (metabolite data) and **X** is an *n*-observation by *p*-variable matrix of predictors (the set of spectral data with *p* wavebands), PLSR aims to decompose **X** into a set of *A* orthogonal scores such that the covariance with corresponding **Y** scores is maximized. The X-weight and Y-loading vectors that result from the decomposition are used to estimate the vector of regression coefficients, **β**_*PLS*_, such that **Y** = **X** **β**_*PLS*_ + ε where ε is an *n* × 1 vector of error terms.

The R package “pls” (Mevik and Wehrens, 2007) was used for PLSR in this study. Separate PLSR models were constructed with each metabolite as a single dependent variable (**Y**) and the 2151 spectral channels as the independent variables (**X**). To choose the appropriate number of factors for each model (*A* from above), leave-one-out (LOO) cross validation was used to estimate root mean squared error (RMSECV) for models fit with zero through 12 factors (linear combinations of the spectral channels), and the model that resulted the smallest RMSECV was selected for further analysis and reporting. To evaluate models with independent data, efforts focused on randomly subdividing the data set into four groups without replacement. Models were fit with data from three of the groups (training set of 33 samples), while data from the fourth group was used for model testing (validation set of 11 samples), and the process was iterated such that each group of samples was used for model testing one time. Modeling results from this independent testing methodology (“4-fold CV models”) were compared to results from fitting the model with the entire data set (“full dataset models”). The goodness-of-fit (*R*^2^) for the “full dataset models” and the predictability (*Q*^2^) for the “4-fold CV models” were calculated as follows:

$$\begin{array}{l} R^{2}=1-RSS/TSS\\ Q^{2}=-PRESS/TSS \end{array}$$

where *RSS* is the residual error sum of squares, *PRESS* is the predictive residual error sum of squares, and *TSS* the total sum of squares.

Variable importance selection of metabolite models was calculated by ranking the spectral wavelengths (from 1 to 2151) based on their absolute regression coefficient, |**β**_*PLS*_|, with rank 1 for the wavelengths with the highest absolute value. Mean rank numbers of the wavelengths were calculated across groups of metabolites belonging to the same class to obtain metabolic class ranks.

## Results

### Meteorological Conditions and Stress Intensity at the Experimental Site

**FIGURE 1:**
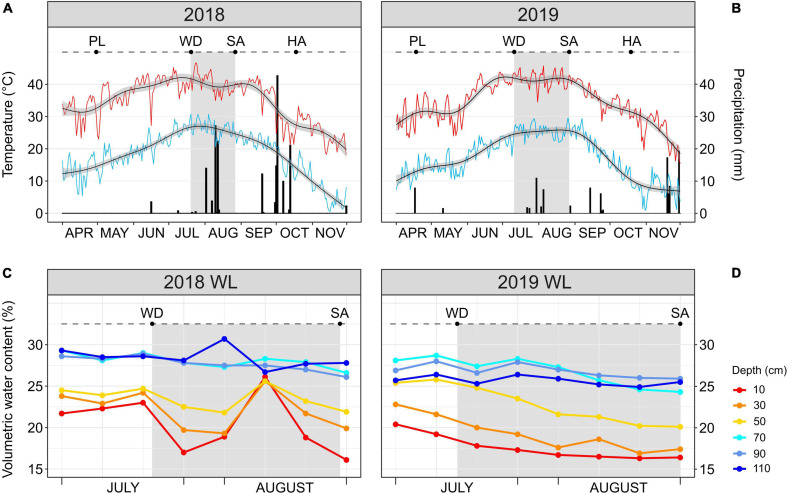
**(A,B)** Daily maximum (red line) temperature, minimum (light blue line) temperature and precipitation (black bars) during the 2018 and 2019 cotton growing seasons. Smoothed generalized linear models (black lines with gray confidence interval) overlay each temperature curve. **(C,D)** Soil moisture probe reads at different soil depths (from 10 to 110 cm) for the water-limited (WL) treatment during July–August 2018 and 2019. In every panel, the stress window during which the WL irrigation regime was applied is highlighted in gray. PL, date of planting; WD, date of start of the WL irrigation regime; SA, date of leaf sampling; HA, date of harvest.

The 2018 and 2019 growing seasons (late April through mid-October) were characterized by a similar temperature trend; however, precipitation was greater in 2018 than in 2019 with a total of 181 and 102 mm, respectively (Figures 1A,B). The same temperature trend applied to the stress windows (highlighted in gray in Figures 1A,B) of 2018 (from Julian days 201 to 239) and 2019 (from Julian days 192 to 239), both occurring during the boll development stage of the crop. The mean high temperature of the two stress windows was higher than 40°C (40.3 ± 3°C in 2018; 41.6 ± 2.3°C in 2019). The 2018 stress window was characterized by a total precipitation of 69 mm which was approximately three times higher than in the stress window of 2019 (24 mm). Additionally, three significant rainfall events (precipitation > 10 mm) occurred in August during the 2018 stress window, one with more than 10 mm of precipitation (Julian day 214) and the other two with more than 20 mm each (Julian days 222 and 224) (Figure 1A). During the 2019 stress window, the only significant rainfall event occurred at the end of July (Julian day 211) with approximately 10 mm of precipitation (Figure 1B).

The occurrence of three consecutive significant rainfall events during the 2018 stress window increased the soil volumetric water content in the WL plots, as quantified by the soil moisture probes (Figure 1C). This increase took place in the middle of the 2018 stress window and involved only the shallow soil layers (10–50 cm), with increases in volumetric water content (%) to the same level as of the deep soil layers (70–110 cm) before decreasing again. Different from 2018, the 2019 stress window saw the cotton accessions grown in the WL plots subjected to a more continuous and increasing water deficit stress with the shallow soil layers always having a lower water content than the deep ones (Figure 1D).

### Effect of Stress on Fiber Yield and Quality

Water-limited plots displayed a marginally significant (*P* = 0.05) mean lint yield reduction compared to WW plots in 2019, but not in 2018 (Table 1). Despite the marginal level of significance, it is noteworthy that a difference in lint yield was detected considering that the trait was calculated based on only 25 randomly collected bolls per plot (date of harvest, HA, is indicated in Figures 1A,B) and not on the entire plot yield. Among the fiber quality traits, Mic, a measurement of fiber fineness and maturity, showed a highly significant (*P* < 0.0001) stress-induced mean reduction in 2019 but not in 2018. Similarly, fiber Str was significantly (*P* < 0.001) reduced by the stress treatment in 2019 only. The only fiber quality trait displaying a significant (*P* < 0.01) stress-induced effect in 2018 was UI, even if the reduction was minimal. Stress-induced UI differences were not observed in 2019 (Table 1).

**TABLE 1:** Fiber traits evaluated under well-watered (WW) and water-limited (WL) conditions in 2018 and 2019 in the 22 cotton accessions.

Fiber trait	Year	Treatment	Value^a^	Treatment effect^b^
Lint yield^c^	2018	WW	37.9 ± 8.3	n.s.
		WL	38.0 ± 8.8	
	2019	WW	41.0 ± 7.8	*P* = 0.05
		WL	39.7 ± 8.2	
Micronaire (Mic)^d^	2018	WW	5.1 ± 0.5	n.s.
		WL	5.1 ± 0.6	
	2019	WW	5.3 ± 0.4	*P* < 0.0001
		WL	4.9 ± 0.4	
Upper-half mean length (UHM)^e^	2018	WW	1.03 ± 0.12	n.s.
		WL	1.02 ± 0.11	
	2019	WW	1.03 ± 0.07	n.s.
		WL	1.02 ± 0.10	
Length uniformity (UI)^f^	2018	WW	80.9 ± 1.9	*P* < 0.01
		WL	80.7 ± 1.8	
	2019	WW	80.9 ± 2.1	n.s.
		WL	80.8 ± 2.2	
Strength (Str)^g^	2018	WW	28.4 ± 2.9	n.s.
		WL	28.1 ± 3.2	
	2019	WW	28.7 ± 2.3	*P* < 0.001
		WL	27.7 ± 2.1	
Elongation (Elo)^h^	2018	WW	5.9 ± 0.5	n.s.
		WL	5.9 ± 0.5	
	2019	WW	5.4 ± 0.4	n.s.
		WL	5.4 ± 0.4	

*^*a*^Mean ± SD.*

*^*b*^Levels: n.s., not significant; *P* = 0.05; *P* < 0.05; *P* < 0.01; *P* < 0.001; *P* < 0.0001.*

*^*c*^Unit of measurement: grams.*

*^*d*^Unit of measurement: air permeability.*

*^*e*^Unit of measurement: inches.*

*^*f*^Unit of measurement: %.*

*^*g*^Unit of measurement: grams per tex.*

*^*h*^Unit of measurement: %.*

### Analysis of Stress-Induced Leaf Metabolic Changes

A total of 217 (GC–MS: 18; LC–MS: 199) metabolites out of 307 annotated ones (70.7%) showed a significant treatment effect in 2018. In 2019, 451 (GC–MS: 27; LC–MS: 424) metabolites out of the 521 that were annotated (86.6% of the total) were significantly affected by the treatment.

**FIGURE 2:**
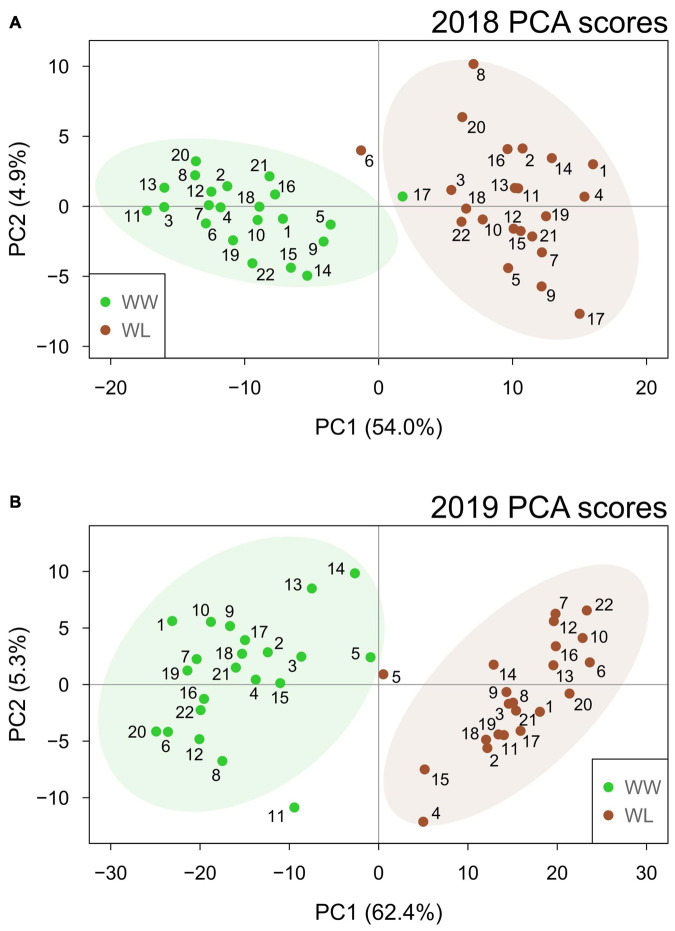
Principal component analysis score plots based on the well-watered (WW, green) and water-limited (WL, brown) values of leaf metabolites for the 22 cotton accessions in 2018 **(A)** and 2019 **(B)**. The percentage of sample variation explained by the first two principal components (PC1 and PC2) is reported in brackets. The number of each sample relates to the “Current study number” column in Supplementary Table 1. Ellipses represent the 95% confidence level for the samples of each treatment.

In both years, PCA (Figure 2) showed that the first two principal components (PCs) effectively separated the two irrigation treatments into their own respective clusters. This separation was more distinct in 2019 when the first two PCs explained a higher percentage of the sample variation (67.7%) than in 2018 (58.9%). Particularly, PC1 alone explained more than 50% of the total variation in both years (54.0 and 62.4% in 2018 and 2019, respectively), and the samples showed a clearer separation based on treatment for this PC. This suggests that PC1 represents the leaf metabolic signature of stress in both years. Different from PC1, PC2 explained a considerably lower percentage of the total variation (4.9 and 5.3% in 2018 and 2019, respectively) and did not separate the samples based on the treatment. In both years, the samples largely overlapped along PC2. Thus, PC2 is likely to represent accession-based leaf metabolic differences within the clusters of WW and WL samples. PC1 and PC2 loading values for 2018 and 2019 are provided in Supplementary Tables 7, 8, respectively. Considering the PCA loading plots of the first two PCs in both years, metabolites did not show any clear clustering based on their main metabolic class (Supplementary Figure 1). Nonetheless, the metabolites with the highest discriminating power (lowest and highest loadings) for PC1, the PC associated with stress-induced differences, mainly belonged to the classes of neutral lipids, polar lipids and, in 2019 only, terpenoids (Supplementary Tables 7, 8 and Supplementary Figure 1).

**FIGURE 3:**
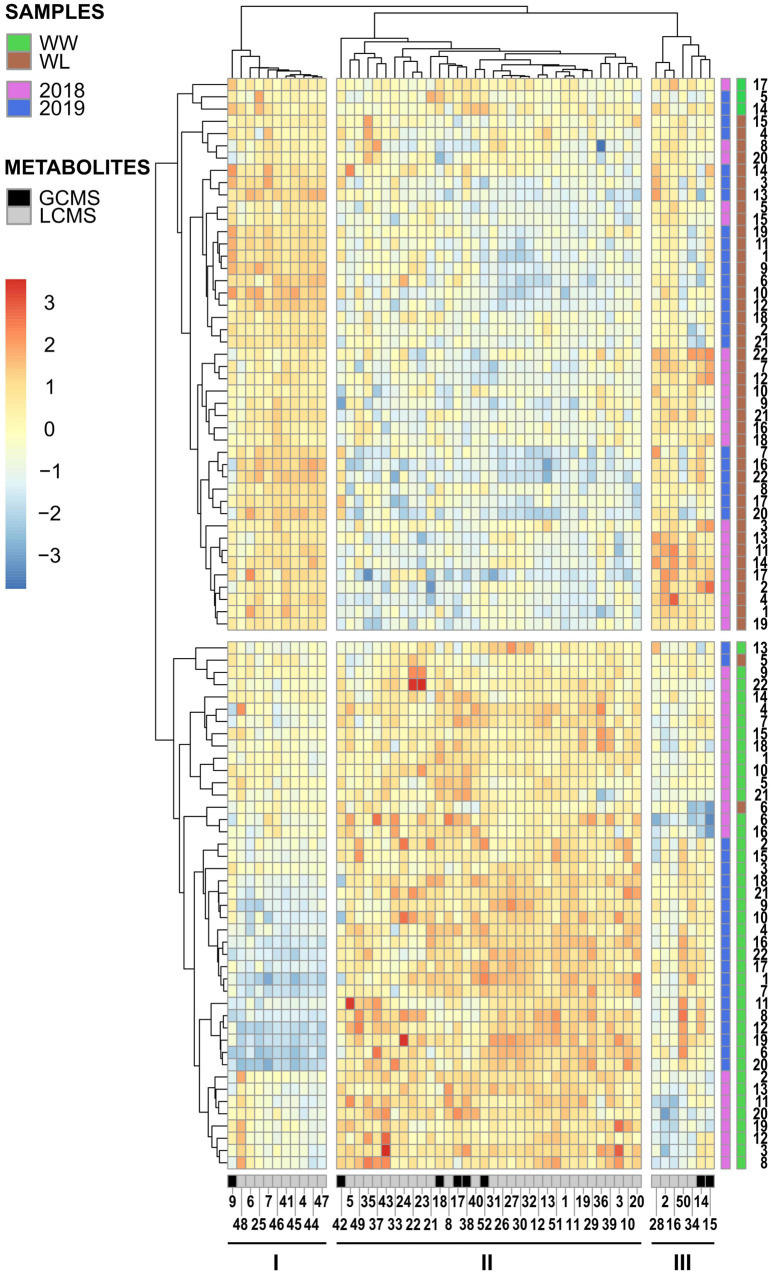
Hierarchical clustering and heatmap of the 52 leaf metabolites identified in common among the 2018 and 2019 leaf samples of the 22 cotton accessions. The scale bar (red to blue) on the left represents normalized intensity of metabolite values. The black and gray bar at the bottom of the heatmap indicates if a metabolite was identified by GC–MS (black) or LC–MS (gray). The first side bar at the right of the heatmap indicates if a sample was collected in 2018 (pink) or in 2019 (blue). The second side bar at the right of the heatmap indicates if a sample was collected from a well-watered (WW = green) or a water-limited (WL = brown) field plot. The metabolite numbers at the bottom of the heatmap relates to the “2018–2019 clustering – Met. Number” column in Supplementary Tables 2, 3. The black lines under the metabolite numbers indicate the three main metabolite clusters (I, II, and III). The sample numbers at the right of the heatmap relates to the “Current study number” column in Supplementary Table 1.

Hierarchical clustering analysis was performed with the metabolites that were identified in both years and displayed a significant treatment effect. These 52 metabolites included ∼24% of the 2018 stress-affected metabolic dataset and ∼11% of the 2019 dataset (Supplementary Tables 4, 5). Hierarchical clustering analysis of the 2018–2019 samples (Figure 3) showed that non-stressed (WW) and stressed (WL) samples of the 2 years formed two main clusters, with only five exceptions (three of these samples resided outside of the 95% confidence intervals of the treatment-induced clusters observed in the PCA score plots, Figure 2). This indicates that samples of the 2 years subjected to the same treatment (2018–2019 WW or 2018–2019 WL) shared more similar leaf metabolite levels than samples of the same year under the two different treatments. The 52 metabolites grouped in three main clusters (I, II, and III in Figure 3). Cluster I and III include metabolites with relatively higher values in WW than in WL samples. Interestingly, cluster I is enriched in neutral lipids, particularly triacylglycerols (TAGs) while cluster III has more polar lipids and carbohydrates and conjugates (Supplementary Tables 4, 5). Cluster II, that includes metabolites with higher values in WL than in WW samples, is the largest of the three clusters and enriched in polar lipids, particularly phosphatidylcholines (PChs), and in amino acids and peptides. Different from cluster I, the neutral lipids present in cluster III are diacylglycerols (DAGs) and not TAGs (Supplementary Tables 4, 5).

To more accurately quantify the effect of abiotic stress on leaf metabolism of the cotton accessions for each year, a FC analysis (WL over WW values) was conducted to identify metabolites that displayed a high mean deviation from non-stressed conditions (the full list of mean FC and significance values for the metabolites is provided in Supplementary Tables 4, 5).

**FIGURE 4:**
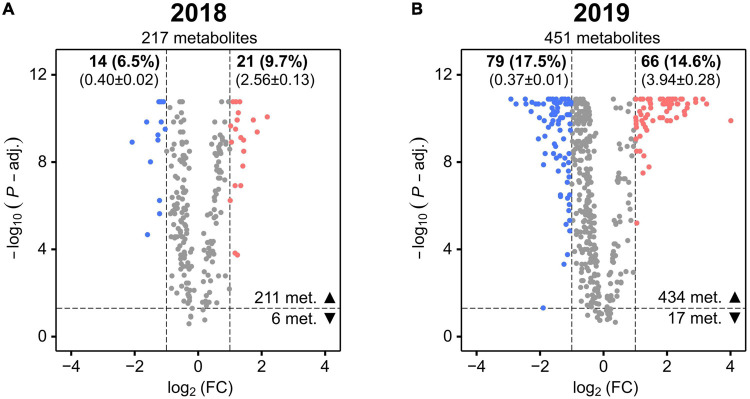
Volcano plots showing the stress-induced significant mean fold changes (FC) of cotton leaf metabolites in 2018 **(A)** and 2019 **(B)**. The horizontal dashed line represents the FDR-corrected Mann–Whitney *U*-test’s *P*-value of 0.05 (*P*-adj.). The vertical dashed lines represent FC <0.5 or >2. For each metabolite, the negative log_10_ of the *P*-value is plotted against the log_2_ of the mean FC. Metabolites with a significant FC decrease or increase are colored in blue and red, respectively (the other metabolites are colored in gray). The total number of metabolites is reported in each plot (under the year) as well as the number of the ones with *P*-value < 0.05 (▲) or *P*-value > 0.05 (▼). The number and percentage (in brackets) of metabolites with *P*-value < 0.05 and with a significant FC decrease and increase is reported in bold font (at top of the plot) together with their mean FC ± SD (below in brackets).

In 2018, only 16.2% (6.5% decrease and 9.7% increase) of the metabolites whose levels were more significantly altered (FDR corrected *P*-value < 0.05) by the treatment displayed also a high mean deviation from non-stressed conditions (Figure 4A). In 2019, the percentage of these metabolites increased to 32.1% (17.5% decrease and 14.6% increase), approximately two times higher than in 2018 (Figure 4B). Additionally, the overall magnitude of these changes was greater in 2019 as indicated by comparing the mean FC of the group of metabolites showing an increase in 2019 (3.94 ± 0.28) with the mean FC of the same group in 2018 (2.56 ± 0.13).

**FIGURE 5:**
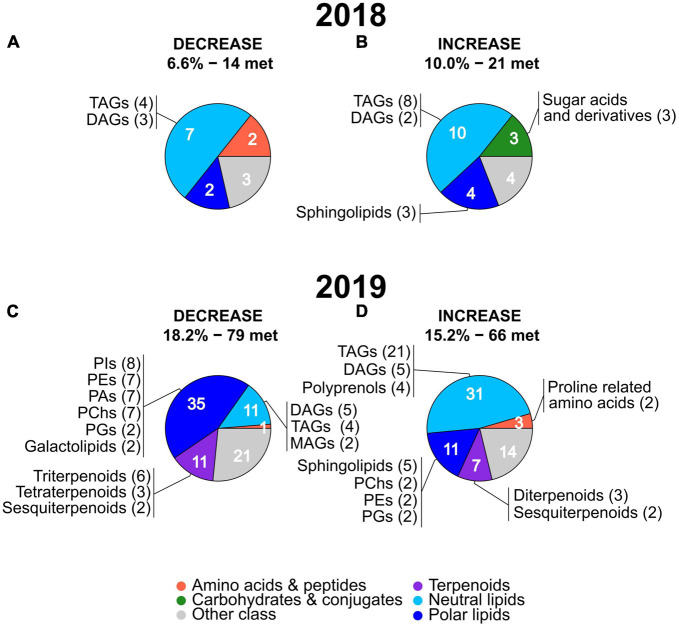
Pie charts showing the main metabolic classes and subclasses of leaf metabolites with the strongest stress-induced mean FC decreases **(A,C)** or increases **(B,D)** in 2018 and 2019. The percentages and number of metabolites (“met.”) of each group (under decrease and increase) are the same as in the volcano plots in Figure 4 and the full lists of these metabolites are provided in Supplementary Tables 2, 3. The number of metabolites present in the main metabolic classes are indicated in white. For each main metabolic class, subclasses including two or more metabolites (with the number of metabolites in brackets) are reported. The name and color of the main metabolic classes are indicated at the bottom of the figure. MAGs, monoacylglycerols; TAGs, triacylglycerols; DAGs, diacylglycerols; Pas, phosphatidic acids; PChs, phosphatidylcholines; PEs, phosphatidylethanolamines; PGs, phosphatidylglycerols; PIs, phosphatidylinositols.

Next, we analyzed if the metabolites showing a high mean deviation from non-stressed conditions in 2018 and 2019 belonged to the same metabolic classes or to different ones. Marked differences were present in the main classes of metabolites with a strong stress-induced decrease in the two years (Figures 5A,C, the full list of the metabolites and their classes are provided in Supplementary Tables 4, 5). In 2018, neutral lipids represented the only abundant (more than two metabolites) and largest class of metabolites (seven) with a similar number of TAGs and DAGs (Figure 5A). In 2019, polar lipids were the largest main class of metabolites (35) showing a stress-induced decrease with a high number of phosphatidylinositols (PIs), phosphatidylethanolamines (PEs), phosphatidic acids (PAs), and a lower number of phosphatidylglycerols (PGs) and galactolipids. The second and third largest main class of metabolites showing a stress-induced decrease in 2019 were terpenoids and neutral lipids, with each class of compounds containing 11 metabolites. Among the 2019 neutral lipids, the most numerous subclasses were DAGs and TAGs followed by monoacylglycerols (MAGs). Among the 2019 terpenoids, the most numerous subclasses were triterpenoids, followed by tetraterpenoids and sesquiterpenoids (Figure 5C).

Focusing on the metabolites showing a strong stress-induced increase (Figures 5B,D), in both years, neutral lipids represented the largest main metabolic class (10 in 2018 and 31 in 2019) with a high number of TAGs followed by a lower number of DAGs. Polyprenols were present only in 2019 among the neutral lipids showing an increase. Similar to neutral lipids, also polar lipids were the second largest main class among the metabolites with stress-induced increased values in both years (4 in 2018 and 11 in 2019). Sphingolipids represented the largest subclass of polar lipids in 2018 and 2019 while PChs, PEs, and PGs were present in 2019 only. A relatively high number of sugar acids and derivatives (main class of carbohydrates and conjugates) was specific to 2018 only while terpenoids, particularly diterpenoids and sesquiterpenoids, and proline related amino acids were present in the 2019 only.

### Vegetation Indices Associated With Plant Physiology and Water Status

The values of some widely used VIs (based on specific reflectance wavelengths), known to be related to the physiological and water status of plants, are reported in Table 2. The NDVI, a general indicator of plant health status (Tucker, 1979; Gamon et al., 1995; Pauli et al., 2016a), was reduced by the stress in both years, but this reduction was significant (*P* < 0.0001) in 2019 only. The sPRI, an indicator of photosynthetic radiation use efficiency (Gamon et al., 1997; Letts et al., 2008), displayed a significant (*P* < 0.05) stress-induced decrease only in 2019 whereas, in 2018, the effect of stress on this index was slightly positive but not significant. Different from the previous two VIs, the CRI, a spectral measurements of total carotenoid content (Gitelson et al., 2002), showed a highly significant (*P* < 0.0001) stress-induced reduction in both years. Finally, we considered a spectral index for the estimation of leaf water content. To this purpose, we selected the ratio between the WI, an indicator of relative water content (Penuelas et al., 1997), and NDVI (WI/NDVI). In both years, WI/NDVI increased under stress (higher values of the index indicate a lower water content) but the increase was more significant in 2019 (*P* < 0.0001) than in 2018 (*P* < 0.01).

**TABLE 2:** Reflectance-based vegetation indices calculated under well-watered (WW) and water-limited (WL) conditions in 2018 and 2019 in the 22 cotton accessions.

Vegetation index	Year	Treatment	Index value^a^	Treatment effect^b^
NDVI^c^	2018	WW	0.837 ± 0.007	n.s.
		WL	0.823 ± 0.011	
	2019	WW	0.823 ± 0.007	*P* < 0.0001
		WL	0.812 ± 0.007	
sPRI^d^	2018	WW	0.499 ± 0.003	n.s.
		WL	0.501 ± 0.005	
	2019	WW	0.507 ± 0.004	*P* < 0.05
		WL	0.504 ± 0.006	
CRI^f^	2018	WW	2.367 ± 0.061	*P* < 0.0001
		WL	1.995 ± 0.096	
	2019	WW	2.378 ± 0.102	*P* < 0.0001
		WL	2.076 ± 0.088	
WI/NDVI^f^	2018	WW	1.235 ± 0.012	*P* < 0.01
		WL	1.259 ± 0.018	
	2019	WW	1.267 ± 0.011	*P* < 0.0001
		WL	1.285 ± 0.012	

*^*a*^Mean ± SD.*

*^*b*^Levels: n.s., not significant; *P* < 0.05; *P* < 0.01; *P* < 0.001; *P* < 0.0001.*

*^*c*^NDVI, normalized difference vegetation index.*

*^*d*^sPRI, scaled photochemical reflectance index.*

*^*e*^CRI, carotenoid reflectance index.*

*^*f*^WI/NDVI, ratio between water index and NDVI.*

### Spectral-Based Models for Metabolite Estimation and Prediction

In 2018 and 2019, the hyperspectral-based “full dataset models” for the estimation of leaf metabolites displayed similar results. Almost all the metabolites (97.2 and 95.6% in 2018 and 2019, respectively) were estimated using one or more linear combinations (latent variables, LV) of the spectral wavelengths (Table 3 and Supplementary Tables 9, 10) suggesting a relationship between chemical composition and reflectance characteristics of the leaves. The strength of this relationship is highlighted by the fact that ∼60% of metabolite variation (mean *R*^2^) was explained by the “full dataset models” in both years. Additionally, the “full dataset models” were able to explain more than 70% variation (*R*^2^ > 0.70) for one third of the metabolites (Table 3).

**TABLE 3:** Summary of hyperspectral-based partial least squares regression (PLSR) models for estimation (full dataset) and prediction (4-fold CV) of leaf metabolite values in the 22 cotton accessions under both well-watered and water-limited conditions in 2018 and 2019.

PLSR model	Parameter	Year 2018 (217 metabolites)^a^	Year 2019 (451 metabolites)^a^
Full dataset models	Estimated metabolites^b^ (nr. – %)	211 (97.2%)	431 (95.6%)
	*R*^2^ (mean ± SD)	0.59 ± 0.22	0.61 ± 0.20
	*R*^2^ > 0.70 (nr. – %)	73 (33.6%)	157 (34.8%)
4-Fold CV models	Predicted metabolites^c^ (nr. – %)	159 (73.3%)	321 (71.2%)
	*Q*^2^ (mean ± SD)	0.40 ± 0.21	0.34 ± 0.17
	*Q*^2^ > 0.50 (nr. – %)	56 (25.8%)	69 (15.3%)

*^*a*^Metabolites showing a significant (*P* < 0.05) change due to the effect of the irrigation treatment.*

*^*b*^Metabolites estimated by full dataset models using one or more latent variables.*

*^*c*^Metabolites predicted by 4-fold CV models in which all the four submodels considered one or more variables and with non-negative *Q*^2^ values.*

Different from the “full dataset models,” the “4-fold CV models” predicted a lower number (73.3 and 71.2% in 2018 and 2019, respectively) of metabolites (Table 3). Overall, this reduction occurred because of a decreased number of leaf samples used for calibration of the submodels with the CV procedure (33 vs. 44) thereby limiting the model’s ability to find relationships between leaf spectra and metabolites. This is noticeable from the submodels that either did not include any spectral LVs (not allowing prediction) or produced negative predictability (*Q*^2^) values (Supplementary Tables 9, 10). Nevertheless, it is interesting that metabolites with negative *Q*^2^ values or that were not predicted by the “4-fold CV models” were characterized by lower mean *R*^2^ values (0.35 and 0.44 in 2018 and 2019, respectively) in their respective “full dataset models” than the metabolites estimated and predicted with both models (0.67 in both years). Therefore, almost all the metabolites that were either poorly or not predicted by the “4-fold CV models” were the ones that already showed a weak relationship with the reflectance spectra in the “full dataset models.” As expected, the mean *Q*^2^ of the metabolites predicted by the “4-fold CV models” was lower (∼40 and ∼34% in 2018 and 2019, respectively) than the mean *R*^2^ of the metabolites estimated by the “full dataset models” (Table 3). However, the *Q*^2^ of the “4-fold CV models” is a true measure of prediction capabilities of the hyperspectral-based models, and it is more relevant than the *R*^2^ of the “full dataset models” that represents the maximum metabolite variation that can be described by the spectral data considering the entire sample set. In this context, it is noteworthy that the “4-fold CV models” predicted, with reasonable accuracy (*Q*^2^ > 0.50), 25.8% (56) and 15.3% (69) of the metabolites in 2018 and 2019, respectively (Table 3).

**FIGURE 6:**
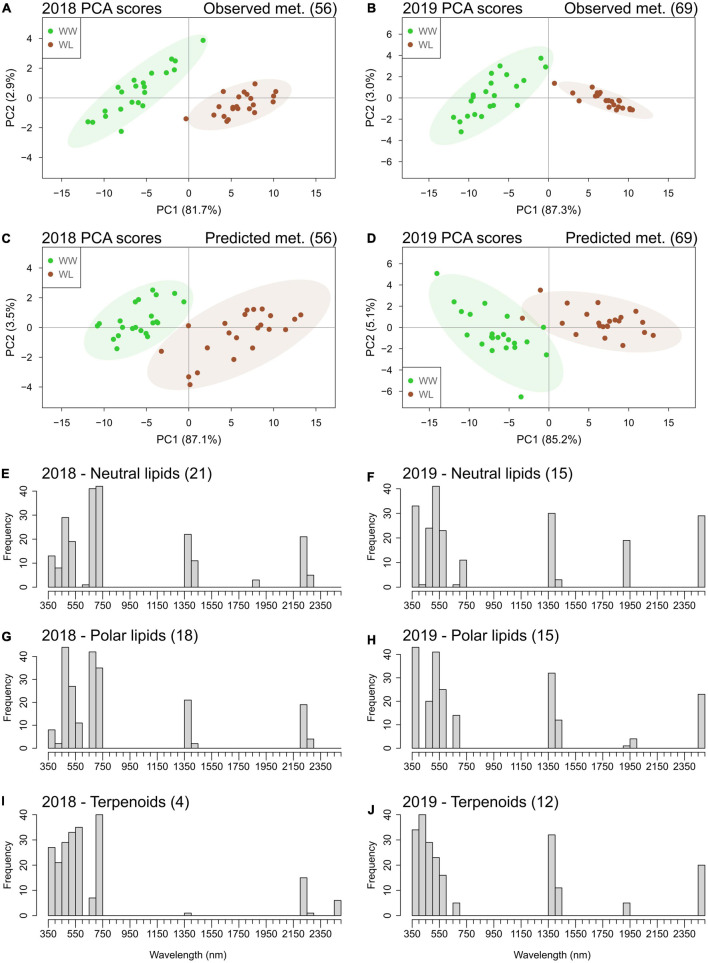
Principal component analysis score plots of leaf samples of the 22 cotton accessions based on observed **(A,B)** and accurately (*Q*^2^ > 0.50) predicted **(C,D)** metabolites (met.) values in 2018 and 2019. The percentage of sample variation explained by the first two principal components (PC1 and PC2) is reported in brackets. Well-watered (WW) leaf samples are in green; water-limited leaf samples are in brown. Ellipses represent the 95% confidence level for the samples of each treatment. Histograms of the frequencies of the top 10% wavelengths selected by the hyperspectral-based models for quantifying metabolites belonging to the classes of neutral lipids **(E,F)**, polar lipids **(G,H)**, and terpenoids **(I,J)** in the 2 years. The number of metabolites in each class is indicated in brackets next to the class name (a full list of these metabolites and the regression coefficients of the wavelengths of their specific models is reported in Supplementary Tables 11, 12).

Interestingly, many of the metabolites that were accurately predicted by the “4-fold CV models” are also in common with the ones that showed a significant stress-induced high mean deviation from control in 2018 (∼54% of the metabolites with high deviation) and in 2019 (∼29% of the metabolites with high deviation) (Figures 4, 5 and Supplementary Tables 11, 12). Therefore, the two sets of metabolites predicted with reasonable accuracy (*Q*^2^ > 0.50) in 2018 (56 metabolites) and 2019 (69 metabolites) could potentially separate the leaf samples based on the treatment. PCA was performed using these metabolites and showed that the first two PCs separated the cluster of WW samples from the one of WL samples using both the observed and the predicted metabolite values, in both years (Figure 6). Alone, PC1 explained more than 80% of leaf sample variation in all the four PCA score plots. On PC1, predicted metabolite values produced a larger overlap between the cluster WW and WL samples (Figures 6C,D) than observed values (Figures 6A,B). This larger overlap was determined by the higher variation in the cluster of WL samples generated by the predicted metabolite values compared with the same cluster generated by the observed values. In summary, PCA results demonstrated that the predicted values of the set of metabolites had only a slightly lower capacity of discriminating the leaf samples based on the treatment than the corresponding observed values.

The metabolites accurately predicted (*Q*^2^ > 0.50) by the “4-fold CV models” predominantly belonged to the classes of neutral lipids, polar lipids, and terpenoids (Supplementary Tables 11, 12). We investigated which regions of the reflectance spectrum were the most informative for the prediction of these metabolic classes. For this analysis, we considered the corresponding “full dataset models” of these metabolites because they maximized the associations between spectral data and compound variation (Table 3). In each year and for each class of metabolites (neutral lipids, polar lipids, and terpenoids), the frequency of the most important (top 10%) wavelengths selected by the models was analyzed (Figures 6E–J). Wavelengths in the ultraviolet–visible (UV–VIS) region (350–750 nm) displayed the highest selection frequencies for all metabolite classes in both years. The frequencies of spectral bands of the UV–VIS region varied between metabolic classes and years. Wavelengths of the near infrared (NIR) region showed no important association with metabolite variation. Interestingly, three spectral bands – 1350–1450, 1900–1950, and 2450–2500 nm – of the short wave infrared (SWIR) region showed high selection frequency for all three classes of metabolites in 2019. In 2018, only one (1350–1450 nm) of the water absorption bands showed high selection frequency in neutral and polar lipids and, to a lesser extent, in terpenoids. Specific for 2018 only was the high selection frequency for wavelengths of the 2200–2300 nm.

## Discussion

A major goal of this work was to analyze the leaf metabolic responses to heat stress and water limitations in a set of genetically diverse cotton accessions grown in the Arizona low desert over two consecutive years.

In both years, the mean high temperature of the stress window was higher than 40°C indicating the presence of strong heat stress (Reddy et al., 1997; Zahid et al., 2016). In our study, the WL treatment was applied during boll development, which is considered a sensitive growth stage to water deficit in cotton (de Kock et al., 1990; Radin et al., 1992). Considering that irrigation was applied with an overhead system and that cotton roots continue to grow in length until the boll development stage (Taylor and Klepper, 1974), it can be assumed that the majority of the root biomass was concentrated in the shallow soil layers during the stress window. Different from 2018, in the 2019 growing season, the shallow soil layers were exposed to prolonged drought stress conditions, strong enough to result in a reduction of fiber yield and quality that was not detected in 2018, when the stress was lower (intermittent drought). The concurring stress-induced reduction of fiber yield and quality in 2019 indicates that the stress intensity in that year strongly altered important biological processes during fiber development.

Similarly, the leaf metabolic profiles of the 22 cotton accessions were altered by water limitations in both years but with this alteration being quantitatively associated with the stronger stress severity in 2019 than in 2018. While PCA results might have been partially influenced by the different sampling time of WW and WL samples (see section “Materials and Methods”), the FC analyses (WL over WW values of metabolites) clearly indicated that stress-induced change of leaf metabolism in 2019 was stronger than in 2018. The analyses of the classes and subclasses of metabolites with the highest deviation from WW conditions revealed that in both years, neutral and polar lipids changed, either increased or decreased, more than other metabolic classes under the combined effect of water limitation and associated heat. Lipids are the main constituents of biomembranes, and membrane lipid remodeling has been described as one of the main plant adaptations in response to abiotic stresses including drought and heat stress (Marchin et al., 2017; Niu and Xiang, 2018; Liu et al., 2019).

In the present work, neutral lipids were the metabolic class with the largest number of metabolites showing a stress-induced increase, particularly TAGs and to a lesser extent DAGs, in both years. In plant vegetative tissues, neutral storage lipids like TAGs are not usually detectable at significant levels in absence of stress, but their accumulation under the effect of other abiotic stresses has been observed in many plant species (Xu and Shanklin, 2016; Lu et al., 2020), including cotton under drought stress (Pham Thi et al., 1985). The accumulation of TAGs in leaves, as lipid droplets in the cytosol or plastoglobules in the chloroplasts, has been linked to their ability to sequester toxic lipid intermediates (e.g., free fatty acids and DAGs) that are generated by membrane remodeling and degradation in response to environmental stress (Yang and Benning, 2018; Lu et al., 2020). Another interesting finding of this study was that, in both years, sphingolipids were particularly numerous among the polar lipids with a high stress-induced increase. These lipids are known for their critical role in maintaining the structural integrity of plant membranes (Gronnier et al., 2018), but they have been also proposed as signaling molecules involved in the abscisic acid-mediated closure of stomata under the effect of drought stress (Ali et al., 2018).

The main classes of metabolites with significant decreases due to stress were different between 2018 and 2019. In 2018, a limited number of metabolites showed stress-induced decreases and most of them were, once again, neutral lipids with a similar presence of DAGs and TAGs. In plant leaves, DAGs are the immediate precursors of TAGs and other polar lipids (Xu and Shanklin, 2016; Lu et al., 2020) and their presence among the metabolites with a strong stress-induced decrease might indicate the synthesis of a different set of lipids deriving from the ongoing membrane remodeling. This hypothesis is supported by the similar number of DAGs among the neutral lipids showing a strong decrease also in 2019.

However, in 2019, polar lipids were the class of metabolites displaying the largest number of strong stress-induced decreases. Many of these polar lipids belong to the subclasses of galactolipids, PGs, PChs, PIs, and PAs, which are the main constituents of the photosynthetic membranes in chloroplasts (Hölzl and Dörmann, 2019). Chloroplast membrane lipid remodeling is an important plant response to abiotic stresses that affects photosynthesis (Niu and Xiang, 2018; Liu et al., 2019). A marked reduction in galactolipids and PChs was observed before in drought stressed cotton leaves and it was associated with sensitivity to the stress (Pham Thi et al., 1985). The reduction of polar lipids that was observed in 2019 may represent a stress-induced imbalance in the biosynthesis/degradation of important components of chloroplast membranes that, in turn, affected their stability and consequently, leaf photosynthesis. Further research incorporating measurements of leaf gas exchange and photosynthetic capacity will be needed to dissect this relationship.

In 2019, the strong increase of two proline related amino acids, leucylproline and proline betaine, both considered osmoprotectants (McNeil et al., 1999; Singh et al., 2015; Sharma et al., 2019) is indicative of leaf dehydration stress. This further supports the presence of a strong alteration of the physiological status of the cotton plants during this year which, in turn, translated into a reduction of fiber yield and quality. Terpenoids were another class of metabolites that showed stress-induced changes (increases and decreases) in 2019 only. Terpenoids are a highly diverse class of volatile compounds with an important role in mitigating the oxidative stress caused by a variety of abiotic stresses, including drought and heat (Vickers et al., 2009; Boncan et al., 2020). The increases and decreases of many terpenoids suggest a stress-induced change in the leaf volatile emission profile of the cotton accessions in response to the pro-oxidative stress conditions and represents a further sign of the severe stress intensity in 2019.

The second goal of this study was to evaluate the associations between leaf reflectance spectra and metabolites. The spectrum of reflected electromagnetic radiation, specifically the wavelengths between 350 and 2500 nm, carries information on plant status, structural properties, and biochemical composition (Mulla, 2013; Araus and Cairns, 2014). These spectral data can be exploited by calculating VIs, which are mathematical relationships between selected wavelengths of the spectrum, that are indicative of general plant status (Jones and Vaughan, 2010). The higher level of stress in 2019, as compared to 2018, was clearly demonstrated by the reduction in fiber yield/quality and the changes of leaf metabolome. The different level of stress intensity between the two years was also detected by three spectral indices: NDVI, sPRI, and WI/NDVI. NDVI, a general measure of crop health (Tucker, 1979; Gamon et al., 1995), showed a significant reduction in 2019, but not in 2018. Reduced NDVI values were observed before in cotton plants exposed to low irrigation regimes in the field and they were considered signatures of stress because of their correlation with lint yield reduction (Sharma and Ritchie, 2015; Pauli et al., 2016a). Similar to NDVI, sPRI, a proxy indicator of photosynthetic radiation use efficiency detected by changes in xanthophylls pigments (Gamon et al., 1997; Letts et al., 2008; Kohzuma and Hikosaka, 2018), displayed a slight significant reduction in 2019 only. This observation further supports the hypothesis of reduced photosynthetic activity of the stressed cotton plants during the 2 year of the field trial as indicated by the reduction in chloroplast membrane lipid components. Finally, in 2019, the highly significant difference in stressed and non-stressed values of WI/NDVI, an indicator of water content normalized by leaf structural differences (Penuelas et al., 1997; Penuelas and Inoue, 1999), suggested a stronger leaf dehydration than in 2018. This finding matches with the increase of proline-related osmoprotectants in leaves of the stressed plants observed in 2019 only.

In the last decade, the reflectance spectra of leaves and plant canopies have proven to be a highly versatile class of phenotypic data (Maes and Steppe, 2019; Yang et al., 2020). Multivariate statistical modeling of hyperspectral reflectance data at leaf or canopy level has been used to predict photosynthetic parameters (e.g., CO_2_ assimilation, stomatal conductance, maximum rate of Rubisco, and PEP carboxylation) and leaf biochemical and structural properties (e.g., pigments, nitrogen, sucrose, and specific leaf area) in many crop species (Ainsworth et al., 2014; Yendrek et al., 2017; Silva-Perez et al., 2018; Meacham-Hensold et al., 2019, 2020; Cotrozzi et al., 2020). However, the use of hyperspectral data for the quantitative estimation of leaf metabolic profiles remains rare with only one other study conducted in durum wheat (Vergara-Diaz et al., 2020).

Our study demonstrates that, in cotton, the overall spectral signature arising from the leaf biochemical composition can be used in PLSR analysis for the accurate quantitative estimation (*R*^2^ > 0.70 for one third of total metabolites) and prediction (*Q*^2^ > 0.50 for the ∼15–25% of total metabolites) of a large number of leaf metabolites. Furthermore, our results demonstrate that stressed (WL) and non-stressed (WW) cotton leaf samples can be efficiently discriminated by using their predicted leaf metabolite profiles, independently from the year and stress intensity of the field trial. Among the metabolites accurately predicted (*Q*^2^ > 0.50) by the hyperspectral-based model, a large number belonged to the classes of neutral lipids, polar lipids, and terpenoids. The analysis of the most important spectral features contributing to the modeling of these classes of metabolites revealed that the UV–VIS region of the spectrum was the most informative for all the classes of metabolites in both years. This suggests that the UV–VIS region of the reflectance spectrum has the highest discriminatory power for the characterization and quantification of metabolites. The same analysis highlighted that bands of the SWIR region, typically associated with water absorption (Curran, 1989), were more important for the prediction of all the three classes of metabolites in 2019 compared with 2018. This result indirectly confirms the presence of a strong dehydration stress only in 2019 (already detected by the increase of proline related amino acids and by the WI/NDVI index) and reveals how the different water concentrations in WW and WL leaves were discriminated by spectral data for the accurate quantification of metabolites. In 2018, another spectral band of the SWIR region, more associated with the absorption of a broad set of chemical compounds such as protein, starch, cellulose, and sugars (Curran, 1989), was always selected by the metabolite models. This may indicate that, in absence of strong differences in water concentration between the WW and WL leaves, the spectral signature of major metabolic compounds was important for quantifying lipids and terpenoids. Therefore, spectral bands of the SWIR region seem to provide information on the plant physiological–biochemical status that is picked by the spectral-based models. However, given the uncertain relationship between spectra and chemical properties, it is challenging to elucidate what specific property or compounds are responsible for explaining metabolic variation as related to spectral data.

The combination of these findings reinforces the idea that multivariate analysis of reflectance spectra detected at the leaf level is a robust methodology for the non-destructive quantification of leaf metabolites. This technique showed the necessary capacity for screening a large number of different genotypes also when grown under the effect of major environmental stressors with different levels of stress severity. Future studies should further explore if the hyperspectral reflectance signal of plot canopies will yield a similar accuracy in the prediction of metabolites. The structural variability of plant canopies, reliance on solar illumination, and reduced resolution due to increasing the distance between hyperspectral sensors (e.g., assembled on manned or unmanned aerial vehicles) and plants adds additional layers of complexity (e.g., interaction between light and leaf inclination and geometry and position of the sensors) in the analysis of the spectral data (Pauli et al., 2016b). This will be an additional step for further increasing the throughput of this technique.

## Data Availability Statement

The datasets presented in this study can be found in online repositories. The names of the repository/repositories and accession number(s) can be found in the article/Supplementary Material.

## Author Contributions

GM conceptualized the questions, analyzed the data, and wrote the manuscript. KT contributed to the execution of the field trial, collected and analyzed the hyperspectral data, and contributed to the manuscript preparation. CB performed the GCMS/LCMS analyses, quantification of compounds, and data analysis. AT assisted with experimental design, execution of field trial, data collection, and preparation of manuscript. LH contributed to the germplasm, assisted with experimental design, and preparation of manuscript. DP conceptualized, designed, and oversaw all aspects of the project including acquisition of funds and manuscript preparation. All authors contributed to the review of the manuscript and all authors have read the manuscript.

## Conflict of Interest

The authors declare that the research was conducted in the absence of any commercial or financial relationships that could be construed as a potential conflict of interest.

## Publisher’s Note

All claims expressed in this article are solely those of the authors and do not necessarily represent those of their affiliated organizations, or those of the publisher, the editors and the reviewers. Any product that may be evaluated in this article, or claim that may be made by its manufacturer, is not guaranteed or endorsed by the publisher.

## Footnotes

1
http://ag.arizona.edu/azmet/

## Supplementary Material

Supplementary Table 1 Name and identifiers of the 22 cotton accessions.

Supplementary Table 2 Repeatability values and significance of fixed effects from the linear mixed models for the fiber traits of the 22 cotton accessions in 2018.

Supplementary Table 3 Repeatability values and significance of fixed effects from the linear mixed models for the fiber traits of the 22 cotton accessions in 2019.

Supplementary Table 4 Annotations and identifiers of the 2018 metabolites showing a significant change due to the effect of the irrigation treatment.

Supplementary Table 5 Annotations and identifiers of the 2019 metabolites showing a significant change due to the effect of the irrigation treatment.

Supplementary Table 6 Repeatability values and significance of fixed effects from the linear mixed models for the spectral indices in 2018 and 2019.

Supplementary Table 7 Loading values of metabolites on principal component 1 and 2 for the 2018 leaf samples.

Supplementary Table 8 Loading values of metabolites on principal component 1 and 2 for the 2019 leaf samples.

Supplementary Table 9 Results of hyperspectral-based PLSR models for the estimation and prediction of metabolite values in 2018.

Supplementary Table 10 Results of hyperspectral-based PLSR models for the estimation and prediction of metabolite values in 2019.

Supplementary Table 11 List of the 56 metabolites accurately predicted by the “4-fold CV models” in 2018.

Supplementary Table 12 List of the 69 metabolites accurately predicted by the “4-fold CV models” in 2019.

Supplementary Data 1 Best linear unbiased estimators of single accessions in the 2 years of the field experiment for all the fiber yield/quality data, metabolites, hyperspectral data, and vegetation indices.
