# Supplementary material for: Assessing Drought and Heat Stress-Induced Changes in the Cotton Leaf Metabolome and Their Relationship With Hyperspectral Reflectance
Source: Front Plant Sci. 2021 Oct 22;12:751868. doi: 10.3389/fpls.2021.751868 (PMC8569624; doi:10.3389/fpls.2021.751868)
Supplement: Supplementary Table 1 — Name and identifiers of the 22 cotton accessions. [file Data_Sheet_1.zip › Supplementary Figure S1.DOCX]

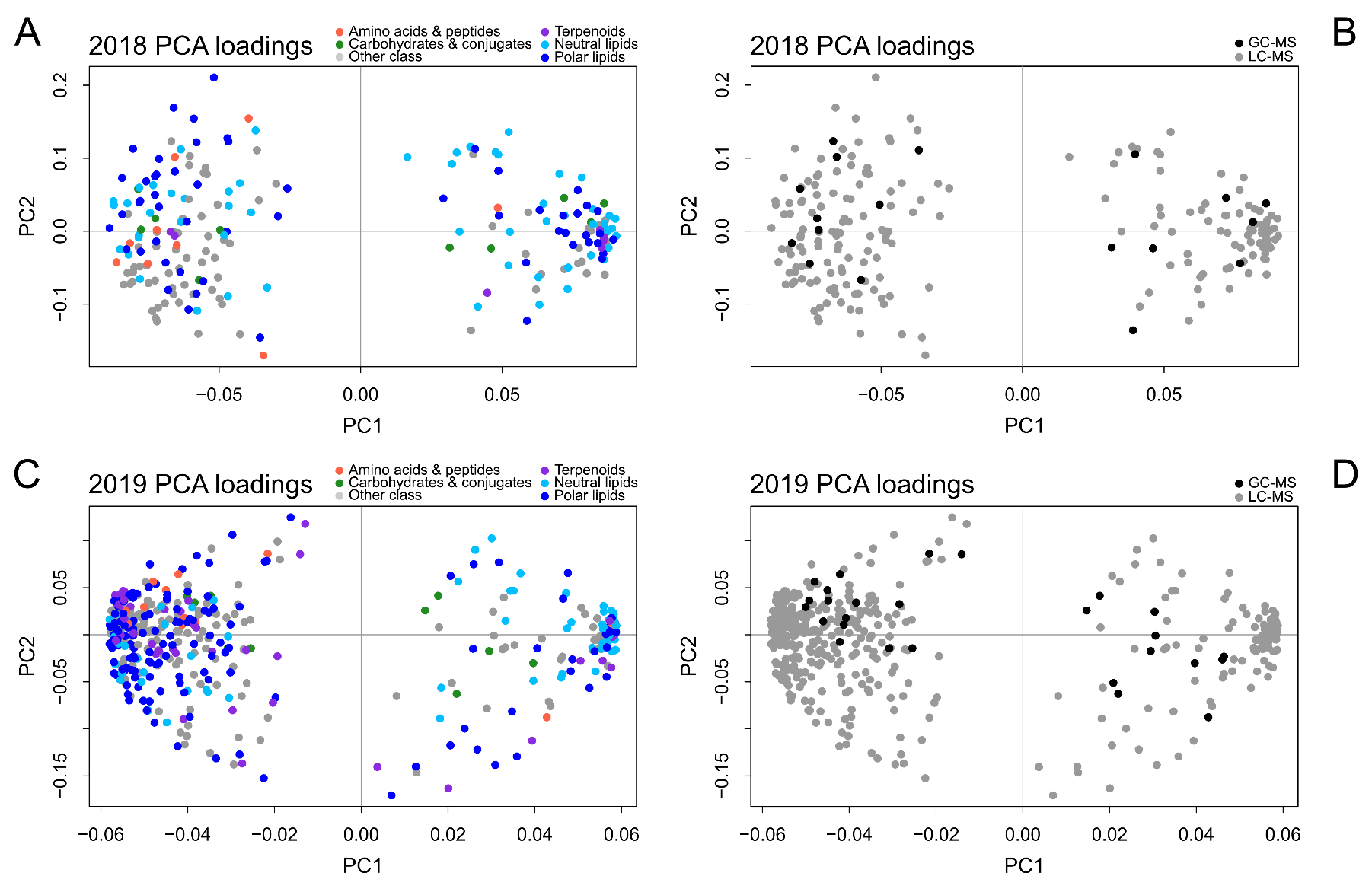


**Figure S1.** Loading plots of leaf metabolites in 2018 (A and B) and 2019 (C and B). (A and C) Metabolites are colored based on their main metabolic class (Amino acid & peptides: red; Carbohydrates & conjugates: green; Neutral lipids: light blue; Polar lipids: dark blue; Terpenoids: purple; Other class: gray). (B and D) Metabolites are colored based on the type of analysis used for their quantification (GC-MS: black; LC-MS: gray).
